# Adrenal adenoma as a cause of atypical psychosis: Presentation, diagnosis, surgical technique and outcome (case report with a brief literature review)

**DOI:** 10.1016/j.ijscr.2021.106187

**Published:** 2021-07-08

**Authors:** Hammam Rasras, Falmata Laouan, Rachid Jabi, Bouziane Mohammed, Noha El Ouafi, Nabila Ismaili

**Affiliations:** a Department of Cardiology, Mohammed VI University Hospital of Oujda, Mohammed First University of Oujda, Morocco; b Department of General and Visceral Surgery, Mohammed VI University Hospital of Oujda, Mohammed First University of Oujda, Morocco; c Laboratory of Epidemiology, Clinical Research and Public Health, Faculty of Medicine and Pharmacy, Mohammed the First University of Oujda, Morocco

**Keywords:** Hyperaldosteronism, Hypertension, Anxiety, Depression, Adrenalectomy, Case report

## Abstract

**Introduction and importance:**

Psychiatric symptoms may be a mode of the revelation of several endocrinopathies, but rarely in primary hyperaldosteronism, which can increase psychiatric comorbidity, as well as cardiovascular risk.

**Case presentation:**

We report a case of a 26-year-old engineer, who suffered from atypical psychosis before being hospitalized for a state of agitation, he presented with high blood pressure and severe hypokalemia. An etiological assessment revealed a right adrenal adenoma, which was afterward resected, with a very good evolution.

**Clinical discussion:**

In this association, a high-level of aldosterone and hypokalemia can be behind these manifestations that present in an atypical form. Treatment is medical by anti-aldosterone or surgical by resection of the adenoma, but the challenge now is to know if we can or not stop psychotropic treatment after the treatment of the adenoma. In our case, the treatment was stopped six months after the resection of the adenoma, with very good outcomes until now.

**Conclusion:**

Despite the high prevalence of psychiatric illnesses, it is always necessary to look for the organic causes that may be behind these pathologies, especially if they are in atypical forms.

**Learning points:**

• The organic aetiologies of psychiatric pathologies are frequent but very underestimated, being able to threaten the vital prognosis (suicide).
• A good detailed clinical examination can point to an organic etiology which, once treated, avoids complications and relapses.
• Its management must be multi-disciplinary.

## Highlights

• Psychiatric pathologies are very numerous, of which the etiologies are diverse. Organic causes are frequent but unfortunately are very underestimated, hence the prognosis is always poor with a great risk of threatening the vital prognosis (suicide)
• Its management must be multi-disciplinary
• A good detailed clinical examination can point to an organic etiology which, once treated, avoids resorting to abusive diagnostic means and can also avoid complications and relapses.

## 1 Introduction

Neuropsychiatric symptoms as representative of endocrinopathies have been previously described in the literature with high co-morbidity [1]. Their prevalence appears to be much higher in Cushing's syndrome, hyperprolactinemia, and thyroid disease [1]. Until now, only a few studies have reported these symptoms in patients with primary hyperaldosteronism (PH). In this mini-review, we report a case of PH revealed by an atypical psychosis, and through it, we aim to explain its pathophysiology and the influence of different therapeutic strategies.

Our case report was written according to SCARE guidelines [2].

## 2 Case presentation

A 26-year-old male patient, engineer as a profession, from north Africa, with no pathological history (In particular, there are not genetic or psychosocial family history, neither drug history), suffered from an atypical psychosis (anxiety, depression, demoralization, decreased energy, decreased concentration, decreased interest in previously pleasurable activities, insomnia, with an irritable mode) for over four months (he had no past or family history of psychopathy). He was treated by a psychiatrist with neuroleptic (Haldol) and anxiolytic (Bromazepam), but without improvement. Then, he was admitted for a state of agitation, requiring injectable neuroleptic and anxiolytic therapy.

On clinical examination, he presented with mania and delusional syndrome, polyuric-polydipsic syndrome and elevated blood pressure. The biological analysis found severe hypokalemia (1.9 mmol/L). Nicardipine in the self-syringe pusher, with injectable and oral potassium supplementation have been initially introduced.

**Fig. 1:**
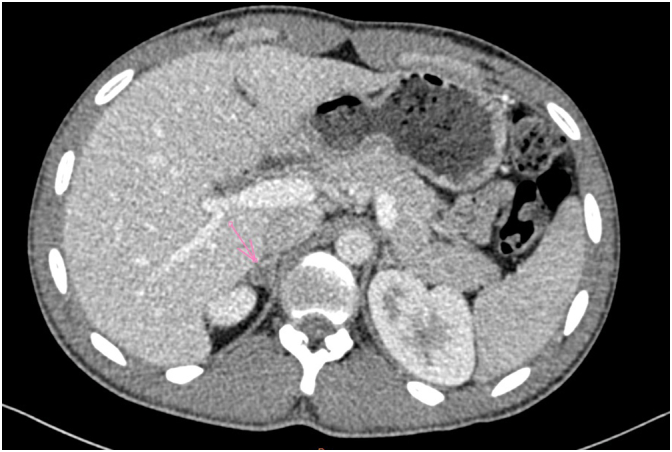
Axial view of abdominal CT scan was performed and showed a right adrenal cortical adenoma.

Faced with this atypical psychosis and these associated signs, an etiological exploration was realized: Brain CT scan, natremia, calcemia, thyroid function test and dosage of urinary methoxylated derivatives were normal. Dosage of activity aldosterone/renin was elevated (161 pmol/mU (normal value < 64)), then an abdominal CT scan was performed and showed a right adrenal cortical adenoma (14 ∗ 11 mm) (Fig. 1/2).

**Fig. 2:**
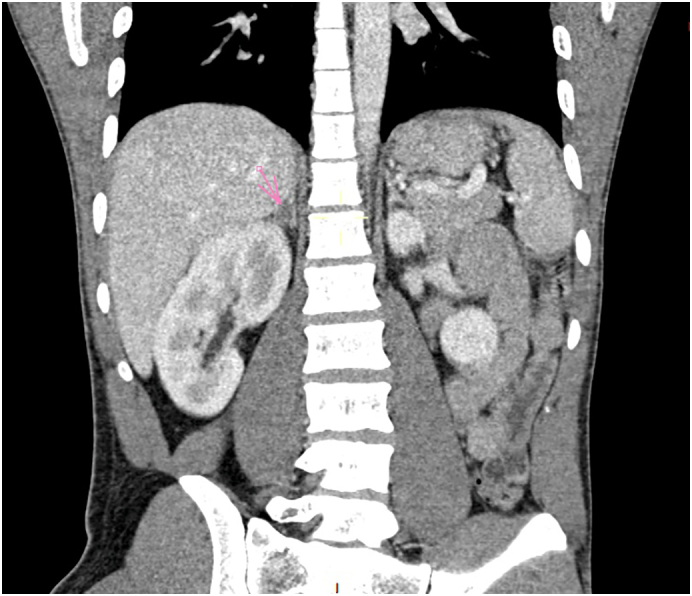
Coronal view of abdominal CT scan was performed and showed a right adrenal cortical adenoma.

**Fig. 3:**
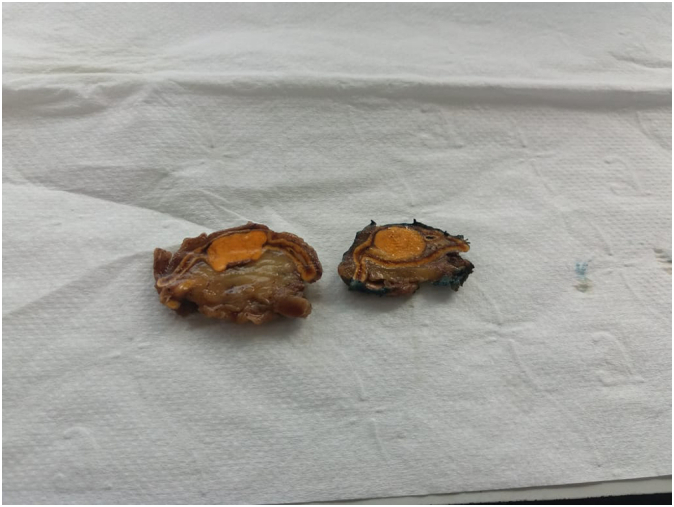
Macroscopic view of the resected mass.

After a multidisciplinary meeting, the patient was six weeks on anti-aldosterone (spironolactone at a progressive dose of up to 200 mg/day) for preoperative preparation, with normalization of blood pressure and kalemia. Then, a Laparoscopic resection of adenoma was performed (Fig. 3) by an experienced professor of visceral surgery with the aid of an assistant professor in the same speciality.

**Fig. 4:**
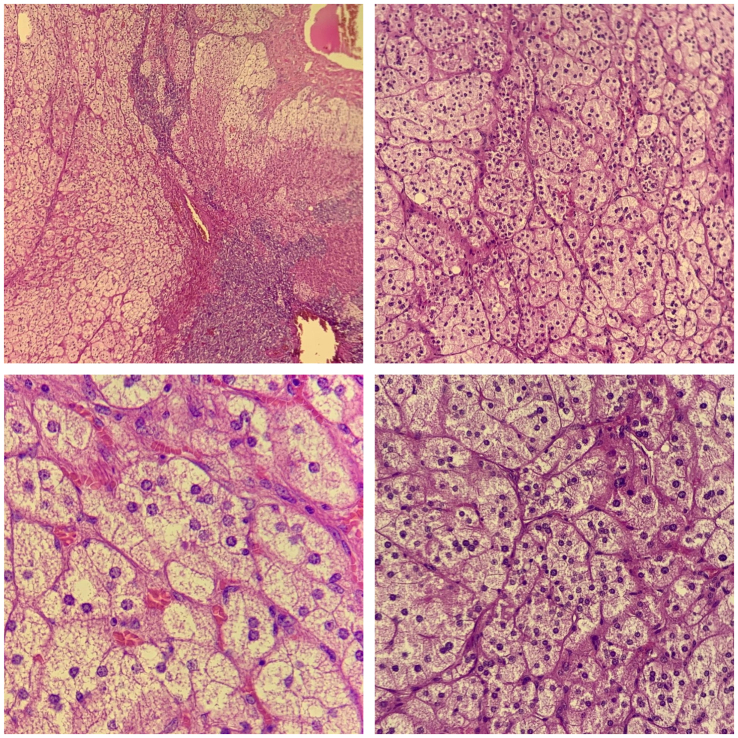
Microscopic views of the resected mass showing an adrenal cortical adenoma without signs of malignancy.

The anatomopathological study that revealed an adrenal cortical adenoma without signs of malignancy (Fig. 4).

Postoperative evolution was good without complications. Spironolactone was immediately stopped with a good control of blood pressure and kalemia. Six months ago, all psychotropic drugs were stopped, and the outcome was favorable; the patient did not present depression, anxiety or a state of agitation, and he was very satisfied of his improvement and he has embarked on his previous job as an engineer.

The patient has an appointment every six months to check for psychiatric symptoms, blood pressure and kalemia.

## 3 Discussion

### 3.1 Descriptive

Primary hyperaldosteronism (PH) or Conn's syndrome (CS) is the most common cause of secondary arterial hypertension, which can be due to adrenal adenoma or bilateral adrenal hyperplasia [3]. PH is usually revealed by treatment-resistant hypertension [4], but it may be also revealed by psychiatric symptoms such as depression, anxiety, irritable mood and impaired quality of life [5] (Table 1).

**Table 1:** Different studies showing the different presentations of psychiatric symptoms in PH-patients.

Psychiatric symptoms	Sonino et al. [10]	Sonino et al. [6]	Apostolopoulou et al. [5]	Our patient
Generalized anxiety disorder	60%	30.4%	50%	Yes
Obsessive-compulsive disorder	10%	13%		No
Panic disorder	10%	8.7%		No
Major depression disorder	10%	8.7%	25%	Yes
Persistent somatization	20%	8.7%		No
Irritable mode	10%	73.5%		Yes
Demoralization	50%	8.7%		Yes

### 3.2 Epidemiology

PH as a case revealed by depression was reported for the first time in 1979 by Malinow [4]. Its prevalence is indeterminate until now. Initially, it was controversial that high blood pressure is responsible for these neuropsychiatric manifestations, but over time, several studies have been performed in this domain. Nicoletta et al. [6] have found that anxiety, depression and irritable mood were more frequent in PH than in essential hypertension (EH), although that the blood pressure values were similar in both cases. They noted also that hypokalemia was only found in patients with irritability. In addition, Emanuele et al. found increased plasma aldosterone levels in patients suffering from depression compared with control subjects [7].

So, all these studies signs that these manifestations are directly because of PH and not anymore due to severe levels of hypertension.

### 3.3 Physio-pathology

Its physio-pathology remains unclear. Aldosterone is known as the major ligand of mineralocorticoid receptors (MR), and it can penetrate the blood-brain barrier. What is hypothetical until now is that excessive aldosterone may induce disturbances in the balance of glucocorticoid receptors (GR) and MR, which may be responsible for symptoms appearance [8]. The place of renin in this physio-pathology is still unknown.

### 3.4 Diagnosis

In our case, the patient suffered from these psychiatric manifestations for a long time; he did not present any neurosensory signs of arterial hypertension, and the detection of his high blood pressure was accidental. It means that the psychiatric manifestations are because of the elevated level of aldosterone and severe hypokalemia, such as mentioned in the studies above.

### 3.5 Treatment and follow up

PH treatment includes adrenalectomy or mineralocorticoid receptor antagonists (MRA). The challenge now is to know which therapeutic strategy to favor in this case. Recent studies in this domain have focused on the post-therapeutic evolution, particularly on improving the quality of life and the disappearance of psychiatric manifestations on the one hand, and on the need to continue anti-psychotic therapy on the other. In returning to the literature (Table 2, Table 3), adrenalectomy is favored over MRA, since that hyperaldosteronism is increasingly accompanied by increased cardiovascular risk, of course without forgetting the side effects of long-period MRA-treatment [1].

**Table 2:** different studies showing the level of effectiveness of surgical treatment.

Treatment type	Sukor et al. [9]	Velema et al. [12]	Apostolopoulou et al. [5]
Unilateral adrenalectomy	Good improvement at 6 months	Normalized of QOL score	→ Decrease in cases of depression (5% vs 25%) → Little decrease in cases of anxiety (44% vs 50%)

**Table 3:** Different studies showing the level of effectiveness of medical treatment.

Treatment type	Ahmed et al. [11]	Velema et al. [12]	Apostolopoulou et al. [5]
MRA therapy	QOL significantly improved at 6 months	Improving in QOL score	→ Decrease in cases of depression (10% vs 25%) → Steady state for anxiety

Sukor et al. [9] showed that the post-therapeutic evolution at three months is much better with adrenalectomy than with medical therapy, while it is almost the same at six months.

In our case, neuroleptic and anxiolytic were gradually stopped three months after the resection of the adenoma, currently and after six months of the resection, our patient presents normal blood pressure, he is hopeful, motivated, and does not present any of the old symptoms.

## 4 Conclusion

Based on this case, after a review of the literature, and because of its poorly understood physio-pathology, Primary Hyperaldosteronism remains a pathology requiring multi-disciplinary management. Here, appears the importance of a good diagnostic approach in psychiatric illnesses, particularly in their atypical forms.

## Consent of patient

Written informed consent was obtained from the patient for publication of this case report and accompanying images. A copy of the written consent is available for review by the Editor-in-Chief of this journal on request.

## Provenance and peer review

Not commissioned, externally peer-reviewed.

## Ethical approval

None.

## Funding

There's no financial support.

## Guarantor

Nabila Isamili, Noha El Ouafi, Mohammed Bouziane.

## Research registration number

None.

## CRediT authorship contribution statement

H. Rasras: Conception, Literature review, Analysis, Data collection, Writing - Review & editing
F. Laouan: Conception, Analysis, Data collection, Writing - Review & editing
R. Jabi: Conception, Software, Writing - Review & editing
M. Bouziane: Conception, Methodology, Supervision
N. El Ouafi: Conception, Methodology, Supervision
N. Ismaili: Conception, Methodology, Supervision.

## Declaration of competing interest

Non-conflicts.
